# The importance of dog population contact network structures in rabies transmission

**DOI:** 10.1371/journal.pntd.0006680

**Published:** 2018-08-01

**Authors:** Mirjam Laager, Céline Mbilo, Enos Abdelaziz Madaye, Abakar Naminou, Monique Léchenne, Aurélie Tschopp, Service Kemdongarti Naïssengar, Timo Smieszek, Jakob Zinsstag, Nakul Chitnis

**Affiliations:** 1 Swiss Tropical and Public Health Institute, Basel, Switzerland; 2 University of Basel, Basel, Switzerland; 3 Institut de Recherches en Elevage pour le Développement, Farcha, N’Djaména, Chad; 4 Veterinary Public Health Institute, Vetsuisse Faculty, University of Bern, Liebefeld, Switzerland; 5 Modelling and Economics Unit, National Infection Service, Public Health England, London, United Kingdom; 6 MRC Centre for Outbreak Analysis and Modelling, Department of Infectious Disease Epidemiology, Imperial College School of Public Health, London, United Kingdom; Wistar Institute, UNITED STATES

## Abstract

Canine rabies transmission was interrupted in N’Djaména, Chad, following two mass vaccination campaigns. However, after nine months cases resurged with re-establishment of endemic rabies transmission to pre-intervention levels. Previous analyses investigated district level spatial heterogeneity of vaccination coverage, and dog density; and importation, identifying the latter as the primary factor for rabies resurgence. Here we assess the impact of individual level heterogeneity on outbreak probability, effectiveness of vaccination campaigns and likely time to resurgence after a campaign. Geo-located contact sensors recorded the location and contacts of 237 domestic dogs in N’Djaména over a period of 3.5 days. The contact network data showed that urban dogs are socially related to larger communities and constrained by the urban architecture. We developed a network generation algorithm that extrapolates this empirical contact network to networks of large dog populations and applied it to simulate rabies transmission in N’Djaména. The model predictions aligned well with the rabies incidence data. Using the model we demonstrated, that major outbreaks are prevented when at least 70% of dogs are vaccinated. The probability of a minor outbreak also decreased with increasing vaccination coverage, but reached zero only when coverage was near total. Our results suggest that endemic rabies in N’Djaména may be explained by a series of importations with subsequent minor outbreaks. We show that highly connected dogs hold a critical role in transmission and that targeted vaccination of such dogs would lead to more efficient vaccination campaigns.

## Author summary

Rabies transmission between dogs and from dogs to humans can be interrupted by mass vaccination of dogs. Novel geo-referenced contact sensors tracked the contacts and locations of several hundred dogs in N’Djaména, the capital of Chad. With the data generated by the sensors we developed a contact network model for rabies transmission dynamics. The model results compared well to incidence data. The model explains the relationship between vaccination campaigns and number of cases better than previous models. Highly connected dogs play a critical role in rabies transmission and targeted vaccination of these dogs would lead to more efficient vaccination campaigns.

## Introduction

The viral disease rabies, transmitted between mammals through bites, is fatal following the onset of symptoms. Although human rabies can be prevented by appropriate post-exposure prophylaxis (PEP), approximately 60,000 people die annually from rabies, mainly in Africa and Asia, [1]. The main source of exposure for human rabies is the domestic dog, so vaccinating dogs is an effective way of reducing rabies transmission among dogs and from dogs to humans [2, 3].

Rabies is endemic in N’Djaména, the capital city of Chad, with an average incidence of one laboratory-confirmed infected dog per week [4]. A deterministic model of rabies transmission predicted that mass vaccination of dogs would be sufficient to interrupt transmission for six years [2]. Vaccination campaigns in dogs were conducted in 2012 and 2013, with both campaigns exceeding 70% coverage [5]. Rabies transmission was interrupted in January 2014 after the second vaccination campaign [3], but there was a resurgence of cases nine months later. Subsequent analyses considered reasons for the quick resurgence, including spatial heterogeneity of vaccination coverage, and dog density; underreporting of cases; and importation. Simulation results from a deterministic metapopulation model suggested that importation was the most likely reason for the case resurgence [6]. Although deterministic models can predict the effect of large scale vaccination campaigns and the overall population dynamics, they do not adequately capture effects of stochasticity in low level endemic settings. This becomes important towards the end of an elimination campaign or upon re-establishment after interruption of transmission [7]. Previous models did not include fine scale heterogeneity at the individual level or the network structure of dog to dog contacts.

The importance of including host contact structure in infectious disease modelling has been highlighted in many studies [8–10]. Theoretical analysis of epidemic processes on graphs has shown that the basic reproductive ratio not only depends on the expected value but also on the standard deviation of the degree distribution of the graph [11] and that on scale-free networks diseases can spread and persist independently of the spreading rate [12]. These theoretical insights led to better understanding of disease transmission dynamics for different diseases, including pertussis [13], influenza [14], severe acute respiratory syndrome (SARS) [15], human immunodeficiency virus and acquired immune deficiency syndrome (HIV/AIDS) [16] and gonorrhea [17], and inspired novel control measures such as acquaintance immunization [18], contact tracing [19] and ring vaccination [20, 21].

Due to the substantial influence of network structure on disease transmission dynamics, many studies have collected data on host interactions. Human contact network models are generally established using contact diaries [22–24], proximity loggers [25–28], video recording [29] or mobile phones [30]. Contacts have also been studied in a wide range of animal species. The most common method for measuring animal contacts is behavioral observation, but other methods such as radio tracking, Global Positioning System (GPS) trackers, proximity loggers or powder marking are also utilized [31].

In the past decades, several rabies models with host contact structure have been published. White *et al.* [32] simulated fox movement pathways using home range size estimates, data from radio tracking and behavioral encounter observations to estimate contact probabilities for different seasons and fox densities. They found that the rabies front set off by an incursion of rabies into a healthy population moved more slowly than in a previous model of homogeneous fox populations. Including contact behavior in the model also resulted in a substantially higher predicted rabies control success rate. Hirsch *et al.* [33] used data from 30 raccoons fitted with proximity loggers to assess properties of the raccoon contact network. Unlike in earlier radiotelemetry studies, they found a highly connected population and discussed possible implications of the social network on the spread of rabies. Reynolds *et al.* [34] used proximity logger data from 15 raccoons to build a contact network model of 90 raccoons and simulate rabies spread. They studied the effects of seasonality, differences in vaccination coverage and impact of behavioral changes in infected raccoons on disease spread. Dürr and Ward [35] used a contact network model of rabies transmission among owned free-roaming dogs in Australia to estimate the impact of a hypothetical rabies incursion from Indonesia. They differentiated transmission within households, between households and between communities. The probability of between household transmission was based on GPS data from 69 dogs, while between community transmission was estimated using questionnaire data. Johnstone-Robertson *et al.* [36] developed a contact network model for rabies in the wild dog population in Australia. They constructed a function for dog contact probabilities, using a wide range of different values to generate contact networks and then implemented a rabies transmission model based on parameters from literature.

However, individual based models of dog rabies transmission in endemic settings are lacking, so this study equipped 300 dogs in N’Djaména with purpose developed geo-referenced contact sensors. This is the first study to collect contact data among dogs as well as the first to integrate contact data from such a large subset of an animal population into a rabies model. The individual based model of rabies transmission we developed includes distance between home locations and a degree distribution fit to a contact network structure of dogs in N’Djaména. We compared our model results to 2016 outbreak data from two quarters of N’Djamena. We examined the re-establishment probability of rabies over different vaccination coverage and compared outbreak probability over time with rabies incidence in N’Djaména from 2012 to 2016. Finally, we investigated the role of individual heterogeneity among dogs and the effect of targeted vaccination strategies.

## Materials and methods

### Contact network data collection

Contact network data was collected in three districts of N’Djaména, Chad, using 300 geo-located contact sensors (GCS) developed specifically for this study. The devices contain Global Positioning System (GPS) modules to track the location and movements of dogs and Ultra-High-Frequency (UHF) technology sensors to measure close-proximity events between dogs. The GCS devices record locations at one minute intervals. For the contact recording, the devices broadcast beacons at one minute intervals and constantly scan for beacons ensuring that no contacts with durations of at least one minute will be missed. Close proximity events were defined as records with a received signal strength indicator (RSSI) of more than -75dBm. Static tests of the devices showed that, independently of the angle between two devices, all contacts closer than 25 cm are registered when signal strength is above that value (S1 Fig).

**Fig 1:**
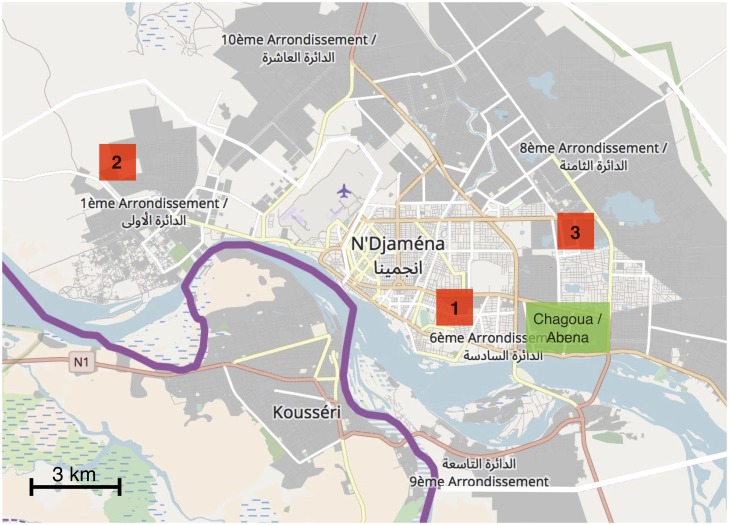
The location of the three study zones (red squares) and Chagoua and Abena quarters (green rectangle) in N’Djamena. The solid purple line denotes the border to Cameroun. The maps were generated using OpenStreetMap contributors.

Collars fitted with the devices were placed on free roaming domestic dogs in three city districts (Table 1, Fig 1) with different dog densities (low, medium and high), that were easily accessible. The zones were chosen to include urban and peri-urban areas. Data were collected during the dry season in December 2016. In the selected districts, all dog-owning households in a pre-defined area of 1km^2^ were identified in order to capture as many of the contacts between dogs as possible, bearing in mind that only contacts between dogs that both wear a sensor can be captured. Dog owners were asked to enroll their pets. Only one dog owner refused to participate in the study. The GCS units remained on the dogs for 3.5 days. After retrieval of the GCS units, dogs were vaccinated against rabies. We excluded study zone 3 from the network analysis due to the low proportion of devices usable for analysis.

**Table 1:** Characteristics of the three study zones. The reason for the discrepancy between the number of dogs and the number of deployed devices is that some collars could not be attached because dogs resisted. In study zone 1 the number of deployed devices was also limited by the fact that we had only 300 devices at our disposal. The discrepancy between deployed and usable devices is due to broken or lost GCS units, battery failure or failure in the data downloading process.

zone	district	location	dog density	number of dogs	deployed devices	devices usable for analysis
1	6	urban	high	328	290	237
2	1	peri-urban	medium	94	80	66
3	8	urban	low	59	41	25

The data from the contact sensors were used to establish an empirical contact network, where the nodes correspond to the dogs and any two nodes are connected by an edge if at least one contact between the two dogs was registered. S2 Fig. shows the number of edges in the empirical network during different subintervals of the study period.

### Rabies cases data

Surveillance of canine rabies in N’Djaména consists of passive reporting of cases confirmed with an immunofluorescence antibody test (IFAT). In 2012, prior to the vaccination campaign, there was, on average, one case of dog rabies per week. After the vaccination campaigns in 2012 and 2013, no rabies cases were reported for nine months. In October 2014, new rabies cases were reported in district number 9, south of the Chari River. In January 2016, the first case north of the river was reported in the Chagoua quarter of district 6 (Fig 1). An additional 6 cases of dog rabies were reported in 2016 in Chagoua and the neighboring Abena quarter.

### Dog population estimates

We simulated rabies incursion into Chagoua and Abena quarters to compare the model results to the outbreak data. Dog population estimates were derived from the 2012 mass vaccination campaign coverage assessment to determine the number of nodes in the network. A total of 2775 dogs were vaccinated during the 2012 campaign in Chagoua, Abena and the neighboring Dembe quarters [5]. A capture-mark-recapture model estimated vaccination coverage in that area at 67%. In a second stage of the campaign, additional dogs were vaccinated in Chagoua, Abena and Dembe. During the latter stage, the proportion of dogs originating from Chagoua and Abena was assessed at 86% of dogs. Assuming that this proportion was the same in the first round, we estimated the dog population in Chagoua and Abena to total 3,500 dogs. This was confirmed through a household survey conducted after the vaccination campaign, which estimated the dog/human ratio to be 1/20. The proportion of ownerless dogs was between 8% and 15% [5]. The total human population in Chagoua and Abena was 72,000 people.

### Network construction

We developed a spatially explicit network construction algorithm to expand the empirical contact network to a synthetic network with more nodes, which allows for more realistic simulations of rabies transmission. When applied to a set of nodes of the same size as the empirical network, this algorithm generated a network with a similar degree distribution. The outbreak probability and size of a rabies transmission model on the empirical and the synthetic network were similar, meaning we captured the features of the empirical network which are relevant for disease transmission in the construction algorithm.

The steps of the algorithm to create the synthetic network are described below. We first create a graph with *n* nodes and zero edges. The number of nodes *n* corresponds to the number of nodes in the empirical network. Each node is assigned a position consisting of *x* and *y* coordinates in a square. The coordinates are sampled using Latin Hypercube sampling. Any two nodes *i* and *j* are connected with a probability *p*_*ij*_ given by *p*_*ij*_ = exp(−*κ*Δ_*ij*_), where Δ_*ij*_ is the Euclidean distance between node *i* and node *j* and *κ* is a scaling parameter. Next a proportion 1 − *τ* of the nodes are selected uniformly at random. For each node *i* in that subset of nodes a number *m* is sampled from a Poisson distribution with mean λ. The node *i* is then connected to exactly *m* other nodes out of all the nodes in the graph. The probability of selecting node *j* into the *m* nodes is given by ${\tilde{p}}_{ij}=\frac{k_{j}}{\sum_{l=1}^{n}k_{l}}$, where *k*_*j*_ is the degree of node *j* and $\sum_{l=1}^{n}k_{l}$ is the sum of the degrees of all the nodes in the graph.

The three scaling parameters, *κ*, *τ* and λ are chosen such that the Kolmogorov distance between the degree distribution of the synthetic network and the degree distribution of the empirical network is minimal. We minimize the Kolmogorov distance by using a gridsearch and confirm the results by minimizing a second metric, the *χ*^2^ distance. The optimal values of the parameters *κ*, *τ* and λ for the two study zones are displayed in Table 2. Larger networks are constructed by choosing the desired number of nodes in the networks and following the steps described above with the optimal values for λ, *τ* and *κ*. The properties of the empirical and the synthetic networks are displayed in Table 3. When optimizing the parameters *κ*, *τ* and λ only the degree distribution of the two networks is taken into account. Therefore, other network properties such as clustering do not necessarily align between the synthetic and the empirical network.

**Table 2:** Optimal values for the three scaling parameters of the network construction algorithm for the two study zones. The optimal values minimize the distance between the degree distributions of the empirical and the simulated networks. The distance is calculated using the Kolmogorov or the Chi-Square metric.

		λ	*τ*	*κ*
Zone 1	Kolmogorov	24	0.75	25
	Chi-Square	23	0.75	25
Zone 2	Kolmogorov	7	0.7	10
	Chi-Square	6	0.7	11

**Table 3:** Properties of the empirical and the synthetic contact networks for the two study zones. For the synthetic network five point summary statistics of 1000 runs of the construction algorithm are displayed.

		empirical	synthetic				
			min	p25	p50	p75	max
Zone 1	nodes	237			237		
	edges	1739	1630	1756	1782	1812	1914
	density	0.0622	0.0582	0.0628	0.0637	0.0648	0.0684
	av. degree	14.68	13.8	14.8	15.0	15.3	16.2
	max. degree	64	39	46	48	50	67
	clustering	0.562	0.129	0.157	0.164	0.172	0.198
Zone 2	nodes	66			66		
	edges	272	238	280	292	305	365
	density	0.125	0.111	0.131	0.136	0.142	0.170
	av. degree	8.24	7.2	8.5	8.8	9.2	11.1
	max. degree	19	19	20	21	29	
	clustering	0.516	0.159	0.230	0.246	0.263	0.339

### Transmission model

We used an individual based transmission model to simulate the spread of rabies in a contact network. All nodes of the network are assigned a status; susceptible, exposed, infective or removed. Nodes infect adjacent nodes with a transmission rate *β* and progress from exposed to infectious and from infectious to removed with average transition periods *σ* and *δ*. For each infected dog the individual incubation period and infectious period is sampled from a Poisson distribution, with mean *σ* or *δ*, respectively. The model ignores birth and natural mortality. The parameter values are displayed in Table 4. The incubation period, *σ*, is chosen from recent literature [37] and fits with the observed time between cases in the incidence data from Chagoua and Abena. The duration of the incubation period is only marginally relevant for our simulations, because it only affects the outbreak duration and not the outbreak probability or size. The infectious period, *δ*, is chosen based on the assumption that a rabid dog in an urban setting would be killed earlier than a natural death from rabies. Our observation that more than two thirds of all samples tested at the rabies laboratory are positive supports the hypothesis that people are likely to recognize the symptoms of rabies since they are less likely to send non-rabid dogs for testing. If people recognise rabies they are more likely to kill rabid dogs. [4]. The transmission rate is chosen using Eq (1). We calculated the mean and variance of the empirical degree distribution, choosing the transmission rate such that *R*_0_ is smaller or equal than 1. We reasoned that rabies is endemic in N’Djaména, with a constant low number of cases and no large outbreaks observed. The transmission rate choice is further supported by the comparison of the simulation results to the outbreak data from Chagoua and Abena.

**Table 4:** Parameters of the rabies transmission model. Time is measured in days.

	Description	Unit	Distribution	Range / Mean
*σ*	incubation period	time	Poisson	90
*δ*	infectious period	time	Poisson	2
*β*	transmission rate	time^-1^	Uniform	[0.015, 0.02]

### Network construction validation

**Fig 2:**
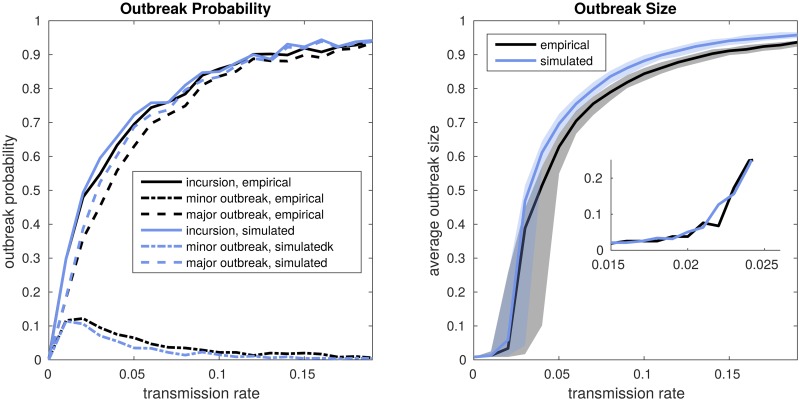
Outbreak probabilities and sizes of the transmission model on the empirical and the constructed network in study zone 1. In each simulation run, one randomly chosen dog is infected from the outside. The simulation ends when there is no more transmission. The incursion probability is the proportion of simulation runs where the number of infected dogs was greater than one. The probability of a minor outbreak is the proportion of simulation runs where more than one dog and less than 1 percent of the population get infected. The major outbreak probability is the proportion of simulation runs where more than 1 percent of the population get infected. The outbreak size is the cumulative proportion of infected dogs over the whole course of the infection. In the left panel the lines correspond to the mean over 1000 simulation runs for each value of the transmission rate. In the right panel the lines correspond to the median over 1000 simulation runs for each value of the transmission rate and the shaded area corresponds to the interquartile range.

We used an individual based transmission model to test whether the properties of the empirical and the reconstructed network lead to similar outbreak probability and size for different transmission rates. The results for 1000 simulation runs of this model on the empirical and the synthetic network are shown in Fig 2. We differentiate between minor outbreaks, which are outbreaks where more than one and less than one percent of the nodes gets infected, and major outbreaks, which are outbreaks where more than one percent of the nodes get infected. Incursions denote all outbreaks where more than one node gets infected and therefore include both minor and major outbreaks. The figure suggests the construction algorithm performs well since the empirical and the simulated network yield similar results in outbreak probability and size. The values of the proportion of simulation runs with outbreaks correspond to the values of the average relative outbreak size, that is the sum of all the final outbreak sizes divided by the number of nodes in the network and the number of simulation runs. This is consistent with the theoretical result that the probability of a major outbreak and the relative size of such a major outbreak are equal [38]. This holds despite the clustering of the synthetic network being higher than in a random graph due to the spatial component of the network construction algorithm. In Fig 2 the outbreak size increases steeply for transmission rate values that are slightly larger than 0.02. This is consistent with the basic reproductive ratio *R*_0_ given by

$$\begin{array}{c} R_{0}=p(\mu +\frac{\text{var}(D)-\mu }{\mu }), \end{array}$$ (1)

where *p* is the transmission probability given a contact and *μ* and var(D) are the expected value and the variance of the degree distribution [38]. In the case of the described network *R*_0_ takes the value of 1 if the transmission rate *β* is 0.02. Since major outbreaks are only possible when *R*_0_ is greater than one, the observed increase of the average outbreak size for values of the transmission probability greater than 0.02 aligns well with the theoretical result, even though not all conditions are met in the case of the described networks.

## Results

### The empirical contact networks

**Fig 3:**
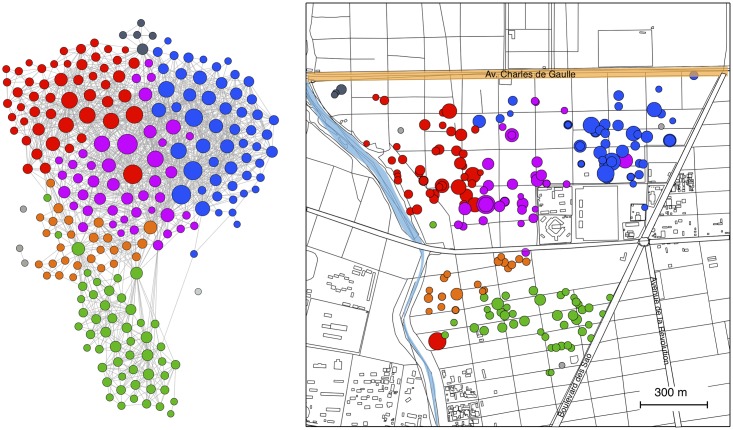
Contact network and home location of the 237 dogs in study zone 1. In the left panel each node corresponds to a dog. The size of the node is proportional to the degree of the node and the color corresponds to the community the node belongs to. Contacts between dogs are shown as grey lines. In the right panel each dot on the map corresponds to a home location of a dog. The colors correspond to the community in the network. The maps were generated using OpenStreetMap contributors.

In study zone 1, the network consisted of 237 nodes and 1739 edges, with an average degree of 15 and and maximal degree of 64. In zone 2, the network consisted of 66 nodes and 272 edges, with an average degree of 9 and a maximum degree of 20. In both zones, nearly all dogs were part of one connected component, that is a sub-graph where any two nodes are connected by a path. The network can be divided into communities using a modularity optimization algorithm [39]. This algorithm optimizes both, the number of communities and the assignment of each node to a specific community, such that the modularity, that is the density of links within communities compared to links between communities, takes the maximum possible value. When the network of study zone 1 is divided into communities using this algorithm it becomes visually obvious that communities mainly consist of dogs which live close together and do not frequently crossrange across roads with traffic (Fig 3). This suggests, that roads with high traffic intensity constitute a functional barrier which substantially reduces contact between dogs residing on either side.

### Comparing simulation results to outbreak data

**Fig 4:**
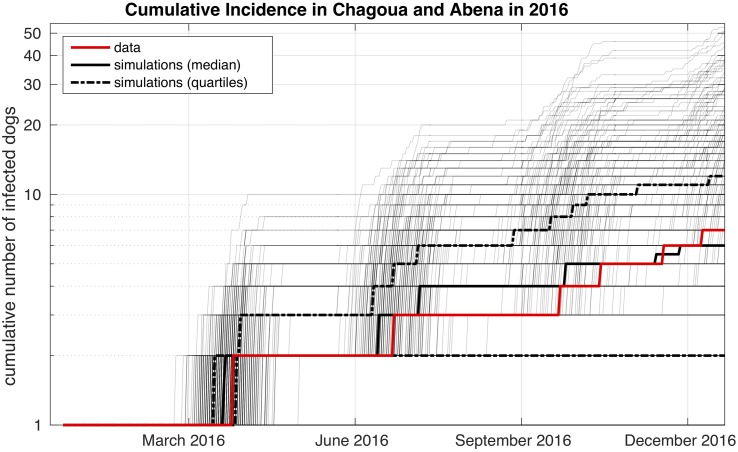
Incidence data and simulation results for the quarters Chagoua and Abena in 2016. The red line is the cumulative number of confirmed rabies cases. The black lines show median and quartiles the cumulative number of cases in simulation runs where the number of rabid dogs was greater than 1. Individual simulation runs are displayed as gray lines.

Rabies was absent from the Chagoua and Abena quarters of N’Djaména for more than a year prior to the outbreak in 2016. The 7 cases were the first to occur north of the Chari River. Chagoua and Abena are virtually separated from other quarters to the west, north and east by main traffic roads and to the south by the Chari River. The area of these two quarters is approximately 4km^2^, and the total number of dogs is estimated to be around 3,500. We simulate the course of the infection after the incursion of one rabid dog. We found that in 450 out of 1000 simulations the chain of transmission was longer than 1, in other words additional dogs get infected. Among these chains of transmission the median of the cumulative incidence of all simulation runs aligns well with the cases observed in Chagoua and Abena (Fig 4). This suggests that the transmission rate in our model is a reasonable choice and that our simulations yield realistic results. Since rabies is often underreported, the true number of cases is likely to be higher than the reported number of cases. We accounted for this in a sensitivity analysis on the reporting probability (S3 Fig). We found that if more than 60% of the cases are reported, the median of the simulations does not differ more from the incidence data than with perfect reporting. The final outbreak sizes are shown in S4 Fig.

### Outbreak probability, size and duration for different vaccination coverages

**Fig 5:**
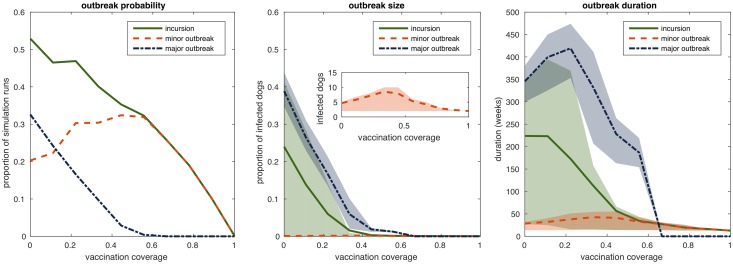
Outbreak probability, size and duration on a network of 4930 dogs for different vaccination coverages. In each simulation run, a proportion of the dogs is randomly assigned the status vaccinated and one randomly chosen susceptible dog is infected from the outside. The simulation ends when there is no more transmission. Simulation runs where more than one dog gets infected are classified as incursion. Simulation runs where more than one dog and less than 1% of the population get infected are classified as minor outbreaks. Simulation runs where more than 1% of the population gets infected are classified as major outbreaks. Incursions include minor and major outbreaks. The outbreak probability is the proportion of simulation runs with outbreaks. The outbreak size is the cumulative proportion of infected dogs over the whole course of the infection. The outbreak duration is the number of weeks until the last infected dog dies. In the left panel the lines correspond to the mean over 1000 simulation runs for each value of the vaccination coverage. In the center and the right panel the lines correspond to the mean over 1000 simulation runs for each value of the vaccination coverage and the shaded areas correspond to the interquartile ranges. The axis of the indented figure in the center panel are the same as in the surrounding figure.

To assess the impact of vaccination coverage on the outbreak probability and size after the introduction of one rabid dog, we constructed a network with a large number of nodes. We considered a 4 × 4 kilometer square and a dog population with the same density as the dog population in study zone 1, which yields a network with 4930 nodes. We ran rabies incursion simulations on that network. The outbreak probabilitiy, size and duration across different vaccination coverage are shown in Fig 5. The probability of a major outbreak, defined as more than 1% of the dog population becoming infected, is substantially reduced when vaccination coverage is above 70%. The probability of minor outbreaks also decreases with vaccination coverage, but only reaches zero with nearly complete vaccination coverage. Even though a minor outbreak, by definition, could affect up to 1% of the population (50 dogs) the simulated average outbreak size is, in fact, very low. This is consistent with the theoretical result that the final number of infected nodes converges to a two point distribution. A proportion of simulation runs stays close to zero whereas the other proportion ends up near the major outbreak size (for an example see S5 Fig). The minor outbreaks, therefore, only capture the short chains of transmission. These chains include, on average, 5 dogs and last approximately 20 weeks, yielding an average number of one infected dog per month which aligns well with the observed endemic situation in N’Djaména [4].

### Time to resurgence

**Fig 6:**
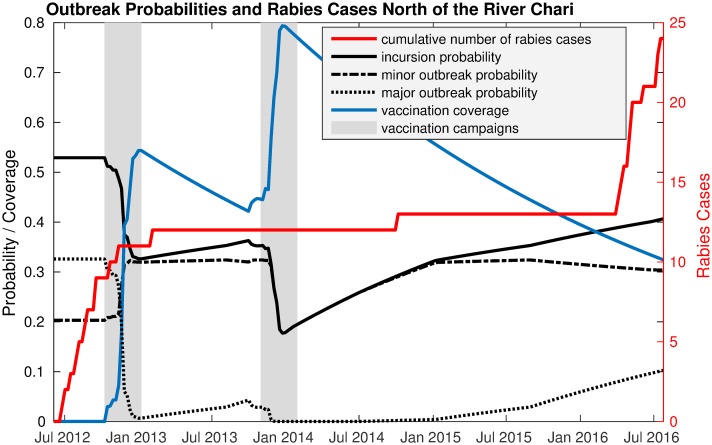
Outbreak probabilities and rabies cases north of the river Chari from 2012 and 2016. For each week the vaccination coverage was calculated using a deterministic transmission model. Vaccination coverages were then translated to outbreak probabilities using the contact network model.

After the vaccination campaigns in 2012 and 2013, no rabies cases were reported north of the Chari River until October 2014. We used a deterministic model [3] to estimate vaccination coverage over time and the contact network model to calculate outbreak probability for the respective coverage. Comparing these probabilities with the incidence data (Fig 6) showed that the first case after the vaccination campaigns could not establish a chain of transmission because the probability for a major outbreak was very low at that time. Later, in February 2016, the respective probability was higher which could explain the subsequent cases.

### Comparing vaccination strategies

**Fig 7:**
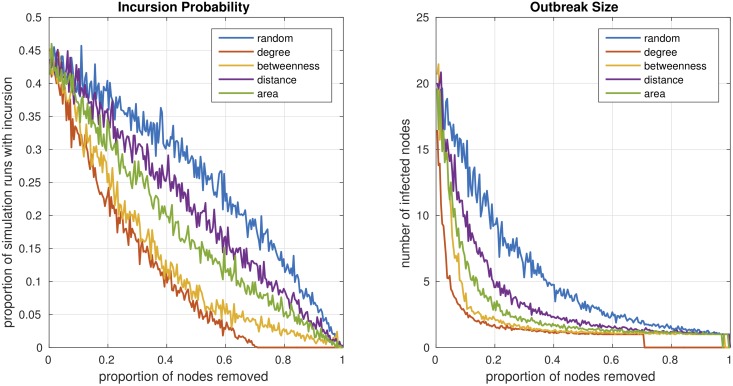
Outbreak probability and size for random and targeted vaccination and different coverages. For each coverage a fraction of nodes is considered as immunized and therefore removed from the network. These nodes are chosen either randomly (blue lines), in descending order of degree (red lines) or descending order of betweenness (yellow line). To simulate the effect of oral vaccination, the probability of a node being immunized was chosen to be linearly proportional to the average distance from the home location (purple line) or the area of the minimal convex polygon fitted into the gps logs (green line). The centrality values of the nodes are recalculated after each node removal. For each strategy and coverage 1000 simulation runs are conducted.

We used the empirical contact network from zone 1 to compare different types of vaccination strategies. Dogs can be vaccinated at random or in a targeted way, based on the contact network structure among the dogs or based on the movements of the dogs. We considered four different ways of targeting dogs: (i) vaccination in order of the degree centrality of the nodes, (ii) vaccination in order of the betweenness centrality of the nodes, (iii) vaccinating each node with a probability that is linearly proportional to the average distance the corresponding dog spent away from the home location of the owner and (iv) vaccinating each node with a probability that is linearly proportional to the area covered by the corresponding dog, where the area was estimated by fitting a minimal convex polygon to the GPS locations of the dog. The outbreak probability and size for each type of vaccination and different coverages are shown in Fig 7. Consistent with previous findings [40, 41] we observed that targeted vaccination reduces the outbreak probability and size more than random vaccination. Targeting nodes by degree yields a lower outbreak probability and size than targeting nodes by betweenness. The betweenness centrality of a node *i* is the proportion of shortest paths between any pair of nodes in the network that pass through node *i*. Nodes with high betweenness centrality are therefore part of many short paths between nodes, which is why removing them affects the global network structure and reduces the size of the largest component, while targeting nodes by degree operates on a local level and reduces the total number of edges more rapidly. In our case, chains on average are short, so the local structure is more important than the global structure. Vaccination based on movement also reduces the outbreak probability and sizes.

### Sensitivity analysis

**Fig 8:**
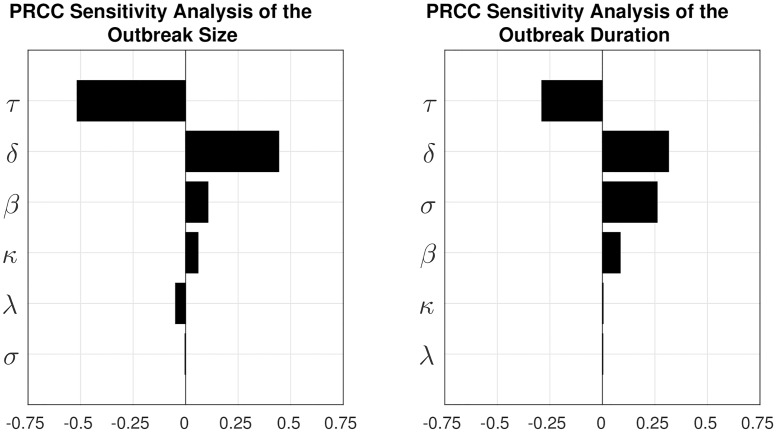
Partial Rank Correlation Coefficient sensitivity analysis of the outbreak size and outbreak duration. The parameter *κ* is a network construction parameter involved in the scaling of the spatial connection. The parameter *τ* is a network construction parameter that alters the proportion of far roaming dogs. The parameter λ is a network construction parameter that alters the mean number of peers of far roaming dogs. The parameter *δ* is the infectious period. The parameter *σ* is the incubation period. The parameter *β* is the transmission rate.

We conducted a Partial Rank Correlation Coefficient (PRCC) sensitivity analysis [42] to assess the impact of the network construction and transmission model parameters on the model output, with ranges as displayed in S1 Table. The results are shown in Fig 8. The most sensitive parameter is *τ*, a scaling parameter of the network construction algorithm. For low values of *τ*, a large proportion of nodes are sampled to connect both to spatially close nodes and any other node in the network. These nodes have a higher degree and betweenness centrality than the other nodes in the network, resulting in an overall larger outbreak size and duration. The remaining two network construction parameters, *κ* and λ, do not have a large effect on the model output. Among the parameters of the transmission model the infectious period, *δ*, is most sensitive. Since the model ignores birth and natural mortality, the incubation period *σ* is only relevant for the outbreak duration and not for the outbreak size. A sensitivity analysis of the outbreak probability, size and duration for different vaccination coverages is shown in S6 and S7 Figs.

## Discussion

This study used empirical contact data to develop a contact network model of dog rabies transmission. We validated the simulation results with 2016 outbreak data from N’Djaména. We used the model to compare the probability of rabies establishment after incursion across different vaccination coverage. We showed that vaccination coverage above 70% prevents major outbreaks, which is consistent with previous findings [3].

In contrast to deterministic models, our individual-based model allowed us to investigate the whole possibility space of outbreak scenarios. Differentiating between minor and major outbreaks revealed that even though the probability of major outbreaks is very low for high vaccination coverage, minor outbreaks can still occur even at nearly complete vaccination coverage. These minor rabies outbreaks consist of approximately 5 dogs, which aligns well with current observations from N’Djaména [4]. The endemicity of rabies in N’Djaména could be explained as a series of rabies introductions with subsequent minor rabies outbreaks, as has been observed in Bangui [43].

We showed that targeting dogs by degree centrality, betweenness centrality or based on their movement substantially increases the impact of vaccination. Targeted vaccination based on betweenness centrality does not perform better than targeted vaccination based on degree centrality. The observation that vaccination by degree performs as well as vaccination according to other network centralities is consistent with previous findings in humans [41]. The degree or betweenness centrality can only be assessed using expensive methods like the tagging with geo-located contact sensors conducted in this study. Such methods cannot be used in routine surveillance. We have shown that vaccination based on movement also reduces the outbreak probabilities and sizes. This might indicate that oral vaccination would be an effective intervention because dogs which cover a lot of territory would be more likely to encounter oral vaccine baits. Oral vaccination has been shown to effectively prevent rabies in dogs [44] and is currently recommended by the WHO as a complementary measure to increase coverage in mass vaccination campaigns [45]. Oral vaccination must be carefully planned with regard to biosafety, for example by assuring that vaccinators retrieve unconsumed baits [46]. It has been successfully implemented to eliminate fox rabies in central Europe [47]. Further consideration of oral vaccination of dogs is warranted based on these results.

We observed a dog population where only a few dogs were not part of the largest component, similar to Hirsch *et. al* [33], who used proximity loggers to reveal a highly connected population in raccons.

In contrast to the raccoon rabies model of Reynolds *et. al.* [34], which concluded that with vaccination coverage of 65% the probability of a large outbreak remains around 60–80%, we noted a substantial drop in the probability of a major outbreak. This might be due to the fact that, while raccoons remain infectious until death from rabies, we assumed that dogs remain rabid for only two days on average because we hypothesized that in an urban setting a rabid dog would be killed by the community. Therefore, major rabies outbreaks could be prevented by rabies awareness and locally reactive interventions.

Unlike Dürr *et al.* [35] who found that even at a vaccination coverage of 70% approximately half the dog population dies from rabies, we found outbreak sizes of less than 1% of the population for high vaccination coverage. This might be due to the fact that Dürr *et al.* considered reactive vaccination after incursion rather than preventive vaccination.

There are several limitations to our study. Our simulations are based on the assumption that rabid dogs stay infective for two days on average, which does not consider the fact, that rabid dogs can be infectious for several days before they show symptoms. Previous models of rabies in wildlife indicated an effect of seasonality on outbreak sizes and durations. Collecting contact data at different times of the year is currently planned, and subsequent analyses will explore the impact of seasonality on contact rates. Dog contacts were only measured for a period of 3.5 days, the extent of battery life. While this observation window is longer than the average infectious period, we cannot be certain that the structure of the network would remain the same when measured for a longer time. Also, contacts with untagged owned dogs and unowned dogs (approx. 8% to 15% of the dog population) were not recorded. Furthermore, we did not include the change of behavior of a rabid animal. However, Reynolds *et. al.* [34] found that assuming a combination of paralytic and furious rabies in the population leads to little quantitative change in the outbreak size.

We found that major rabies outbreaks are unlikely when vaccination coverage is above 70%. Our results suggest that the endemicity of rabies in N’Djaména might be explained as a series of importations with subsequent minor outbreaks. Further investigation of determinants of dog roaming and contact behavior could inform potential targeted vaccination strategies.

## Supporting information

S1 Fig Signal strength of contacts between devices in a static test in N’Djaména. The devices were set up on the ground in a circular arrangement around a central device and contact were recorded for different distances over a period of 1 hour per distance. The colors correspond to different angles from the central device (black dot).
(TIF)

S2 Fig Contacts over time in study zone 1. For each 1 hour interval during the study period, ranging from Saturday 17:00 to Tuesday 7:00, the number of edges in the network is shown. The network for each 1 hour interval was constructed based on all contacts recorded during that interval and an edge was established if at least one contact between the two dogs was registered.
(TIF)

S3 Fig Sensitivity analysis of the simulation results for Chagoua and Abena on the probability of detecting rabid dogs. For each value of the transmission rate (ranging from 0.015 to 0.02 with steps of 0.01) 1000 simulation runs were conducted. Each case in the simulated incidence was randomly assigned as either reported or not reported for different values of the reporting probability (ranging from 0 to 1 with steps of 0.1). The color of each pixel corresponds to the maximum absolute difference between the median of the simulated reported cumulative incidence and the outbreak data.
(TIF)

S4 Fig Final outbreak size of the simulations in the quarters Chagoua and Abena. (TIF)

S5 Fig Final outbreak size of the simulations on a network of 4930 dogs, showing the two point distribution expected from theory. (TIF)

S6 Fig Sensitivity analysis of the outbreak probability, duration and size. The colors correspond to different vaccination coverages. For each vaccination coverage and parameter value the mean of 1000 simulation runs is shown. Simulation runs where more than one dog gets infected are classified as incursion. Simulation runs where more than one dog and less than 1% of the population get infected are classified as minor outbreaks. Simulation runs where more than 1% of the population gets infected are classified as major outbreaks. Incursions include minor and major outbreaks.
(TIF)

S7 Fig Sensitivity analysis of the outbreak probability, duration and size. The colors correspond to different vaccination coverages. For each vaccination coverage and parameter value the mean of 1000 simulation runs is shown. Simulation runs where more than one dog gets infected are classified as incursion. Simulation runs where more than one dog and less than 1% of the population get infected are classified as minor outbreaks. Simulation runs where more than 1% of the population gets infected are classified as major outbreaks. Incursions include minor and major outbreaks.
(TIF)

S1 Table Parameter ranges for the PRCC sensitivity analysis. (PDF)
