# Supplementary figures and images for: The importance of dog population contact network structures in rabies transmission
Source: PLoS Negl Trop Dis. 2018 Aug 1;12(8):e0006680. doi: 10.1371/journal.pntd.0006680 (PMC6089439; doi:10.1371/journal.pntd.0006680)

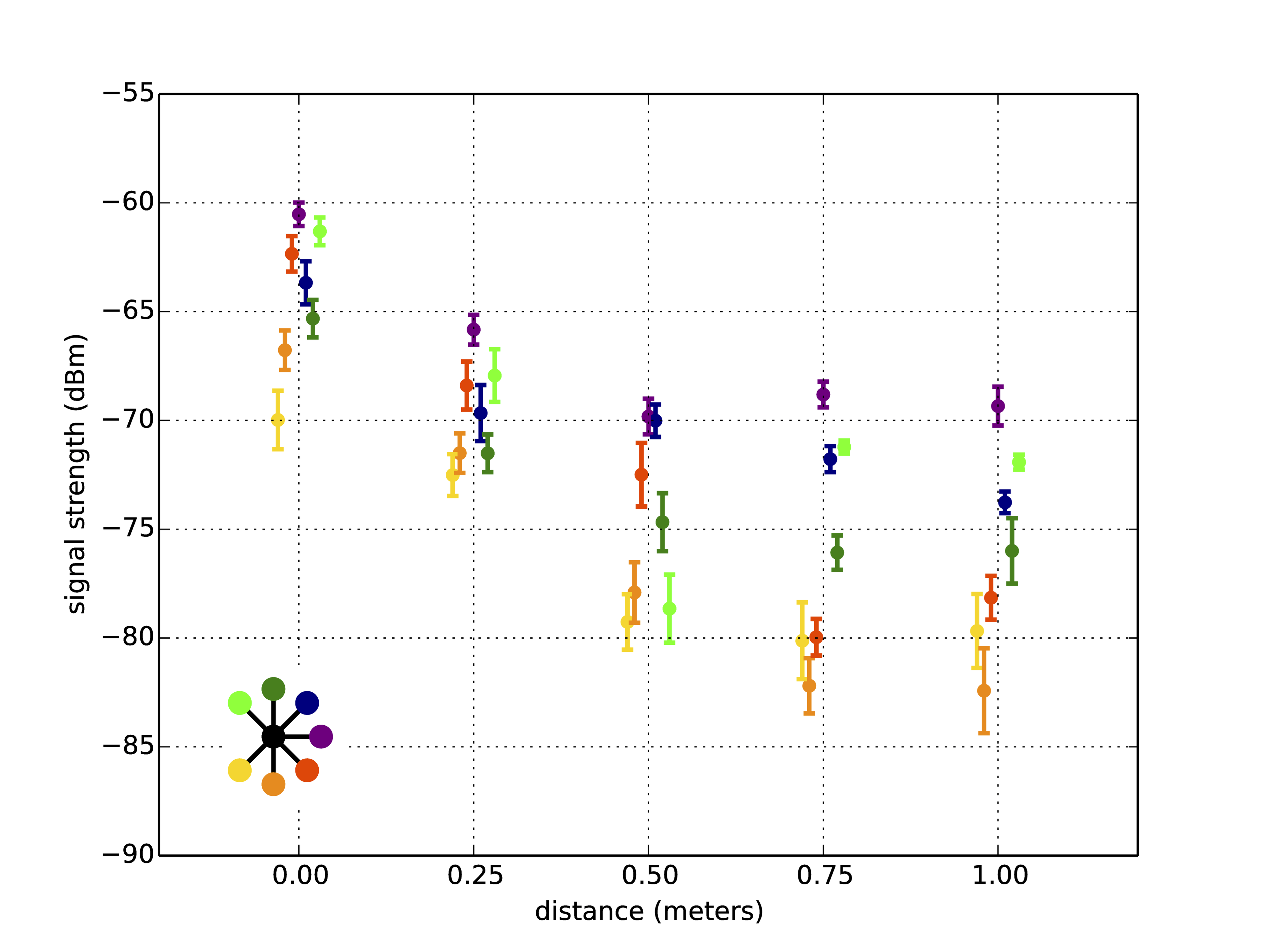

Supplement: S1 Fig — The devices were set up on the ground in a circular arrangement around a central device and contact were recorded for different distances over a period of 1 hour per distance. The colors correspond to different angles from the central device (black dot). (TIF) [file pntd.0006680.s001.tif]

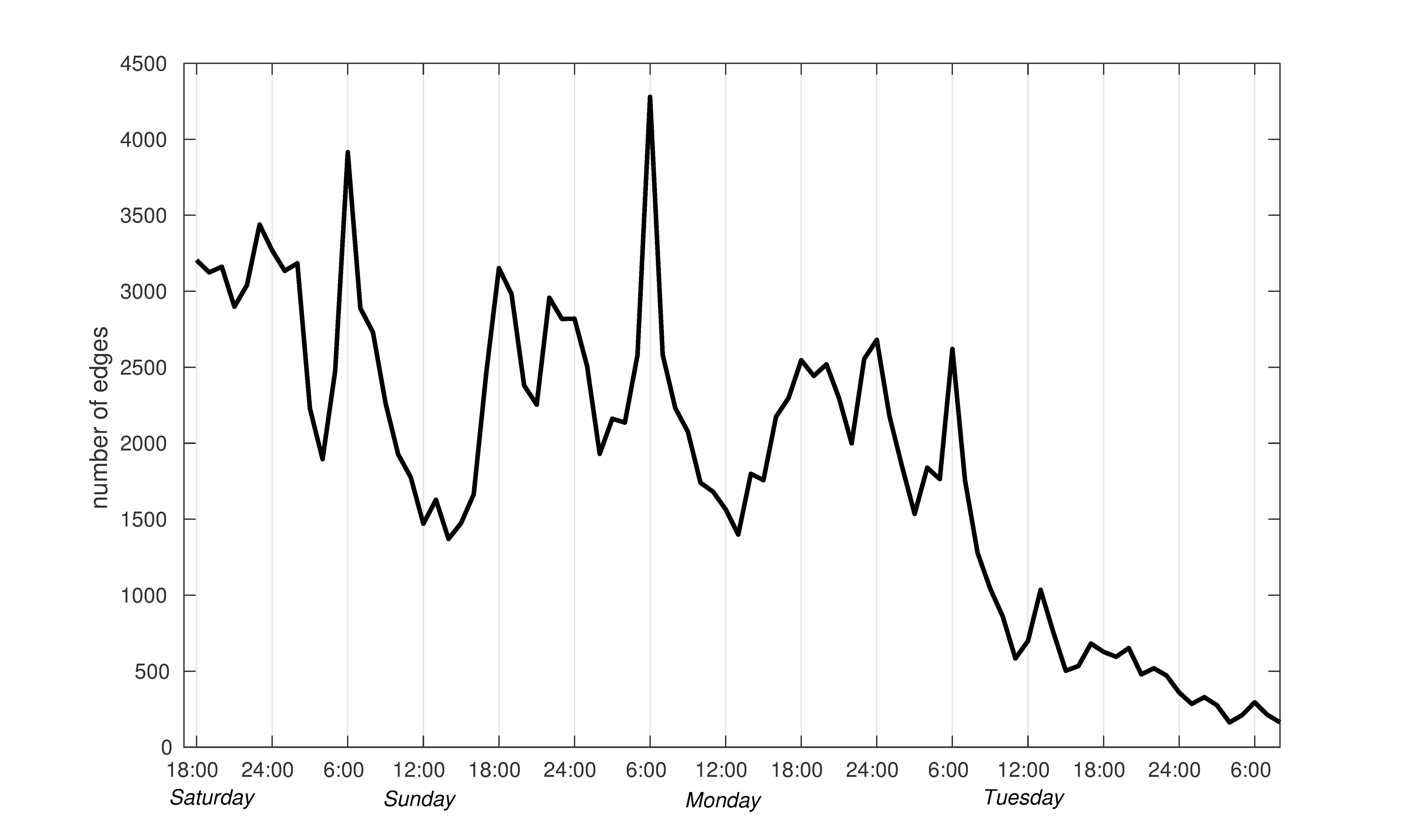

Supplement: S2 Fig — For each 1 hour interval during the study period, ranging from Saturday 17:00 to Tuesday 7:00, the number of edges in the network is shown. The network for each 1 hour interval was constructed based on all contacts recorded during that interval and an edge was established if at least one contact between the two dogs was registered. (TIF) [file pntd.0006680.s002.tif]

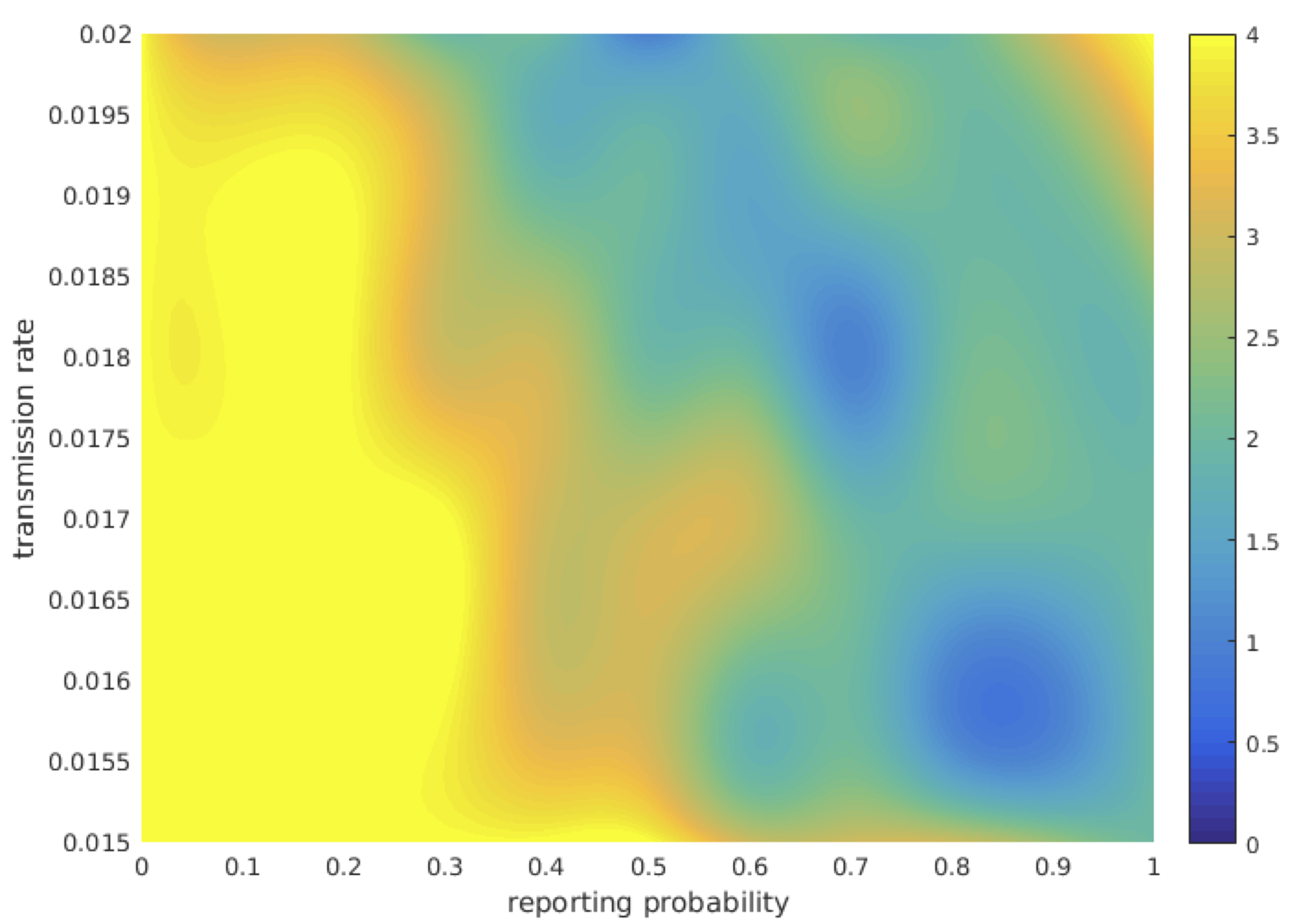

Supplement: S3 Fig — For each value of the transmission rate (ranging from 0.015 to 0.02 with steps of 0.01) 1000 simulation runs were conducted. Each case in the simulated incidence was randomly assigned as either reported or not reported for different values of the reporting probability (ranging from 0 to 1 with steps of 0.1). The color of each pixel corresponds to the maximum absolute difference between the median of the simulated reported cumulative incidence and the outbreak data. (TIF) [file pntd.0006680.s003.tif]

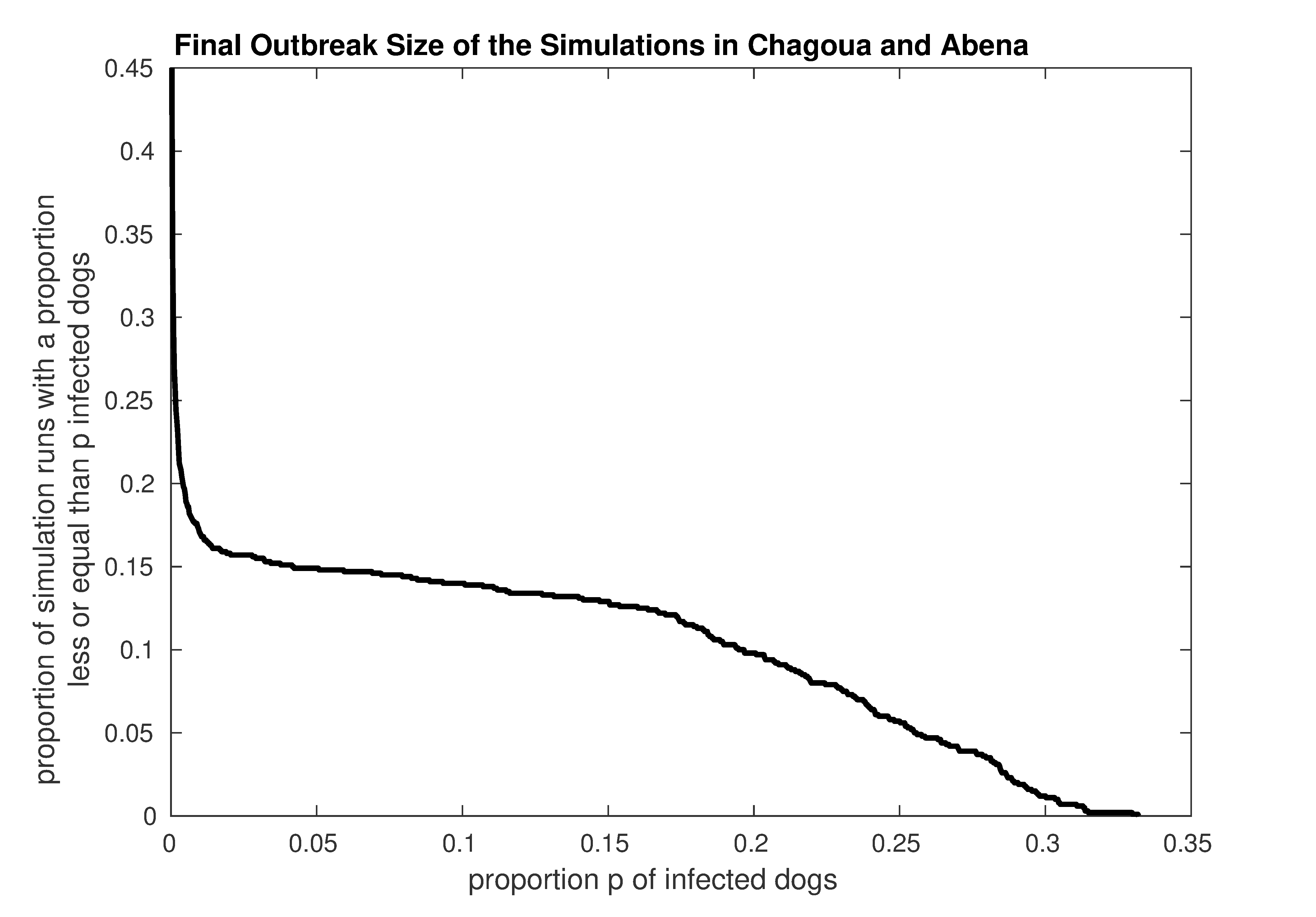

Supplement: S4 Fig — (TIF) [file pntd.0006680.s004.tif]

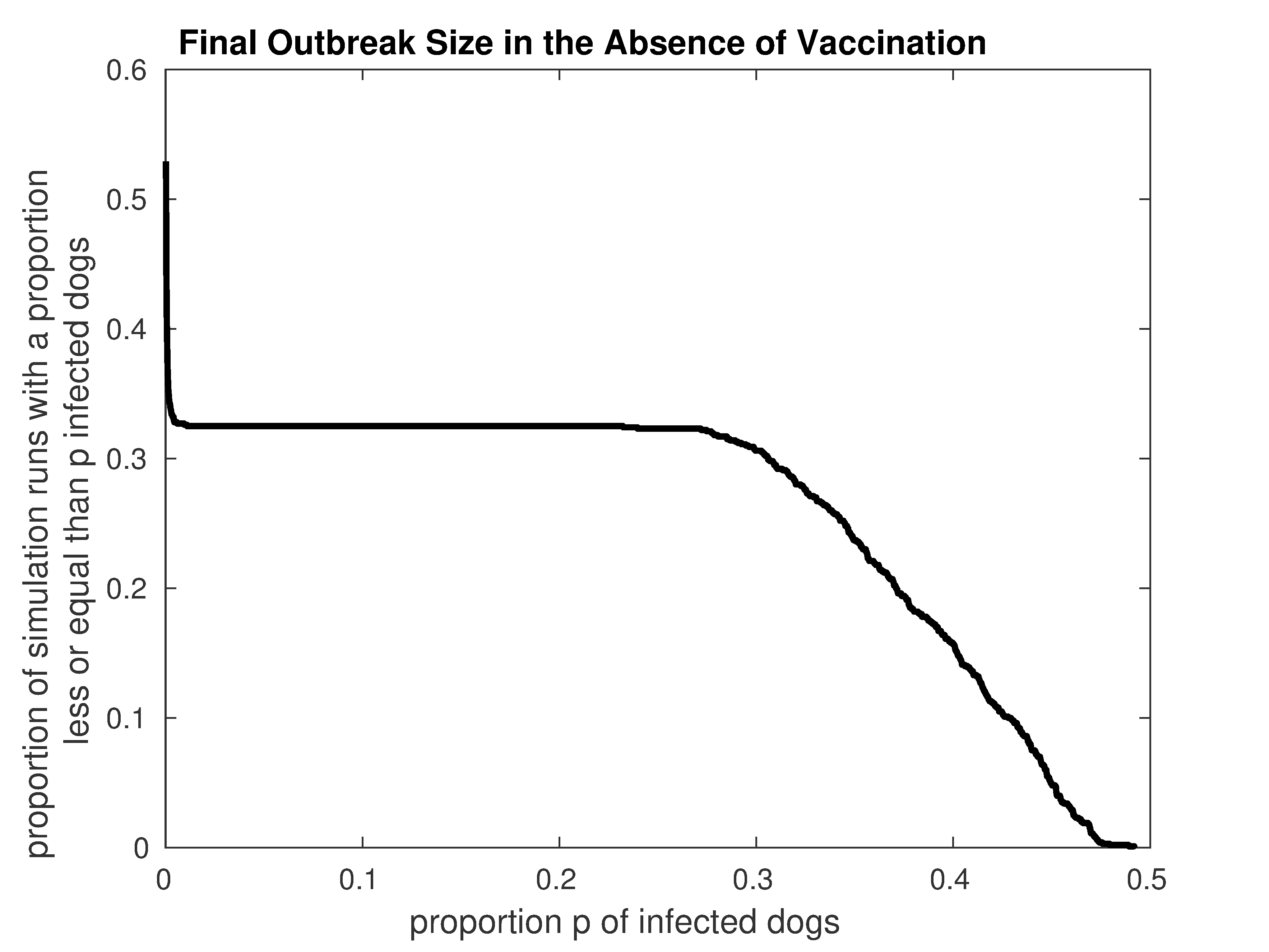

Supplement: S5 Fig — (TIF) [file pntd.0006680.s005.tif]

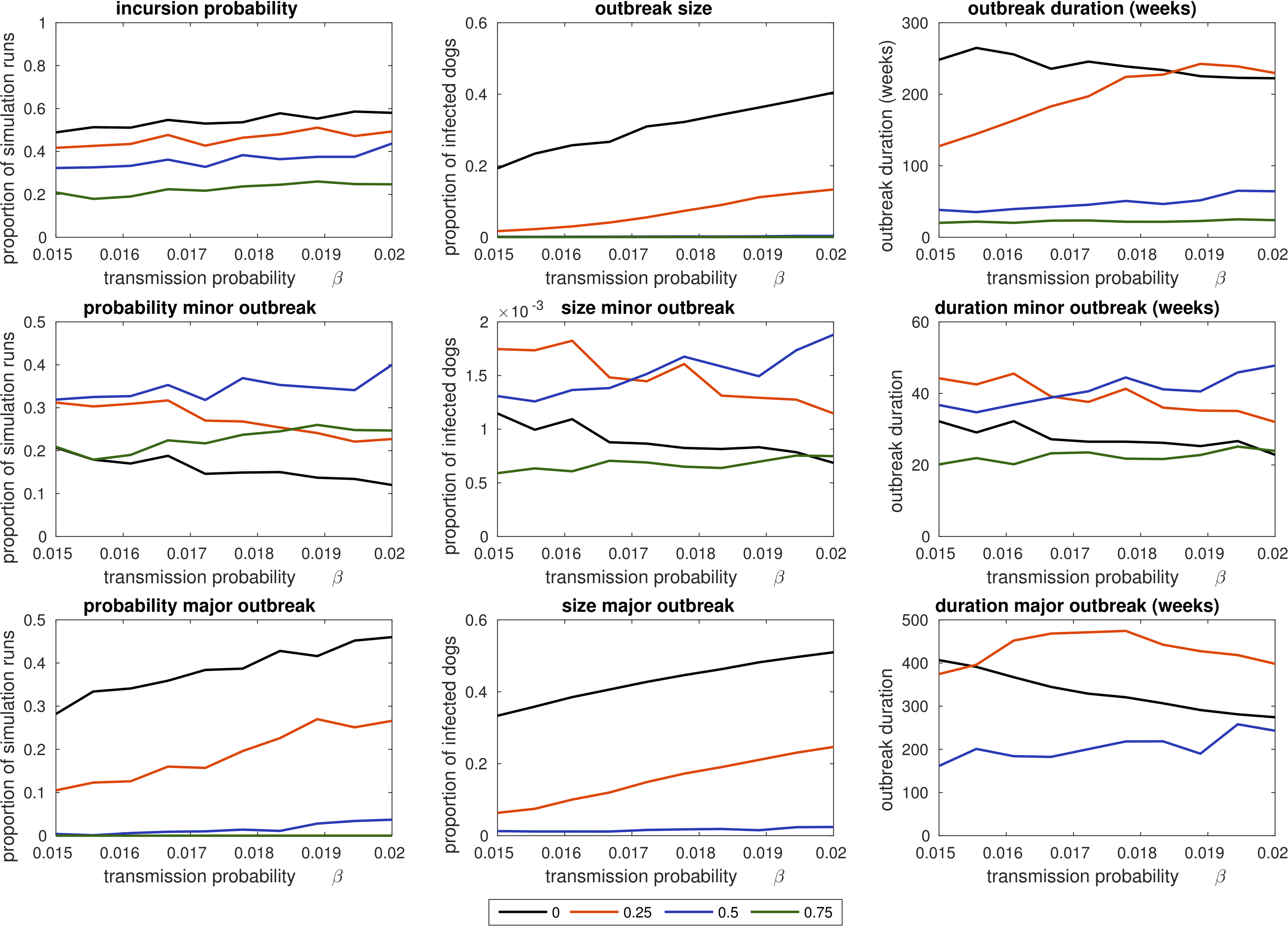

Supplement: S6 Fig — The colors correspond to different vaccination coverages. For each vaccination coverage and parameter value the mean of 1000 simulation runs is shown. Simulation runs where more than one dog gets infected are classified as incursion. Simulation runs where more than one dog and less than 1% of the population get infected are classified as minor outbreaks. Simulation runs where more than 1% of the population gets infected are classified as major outbreaks. Incursions include minor and major outbreaks. (TIF) [file pntd.0006680.s006.tif]

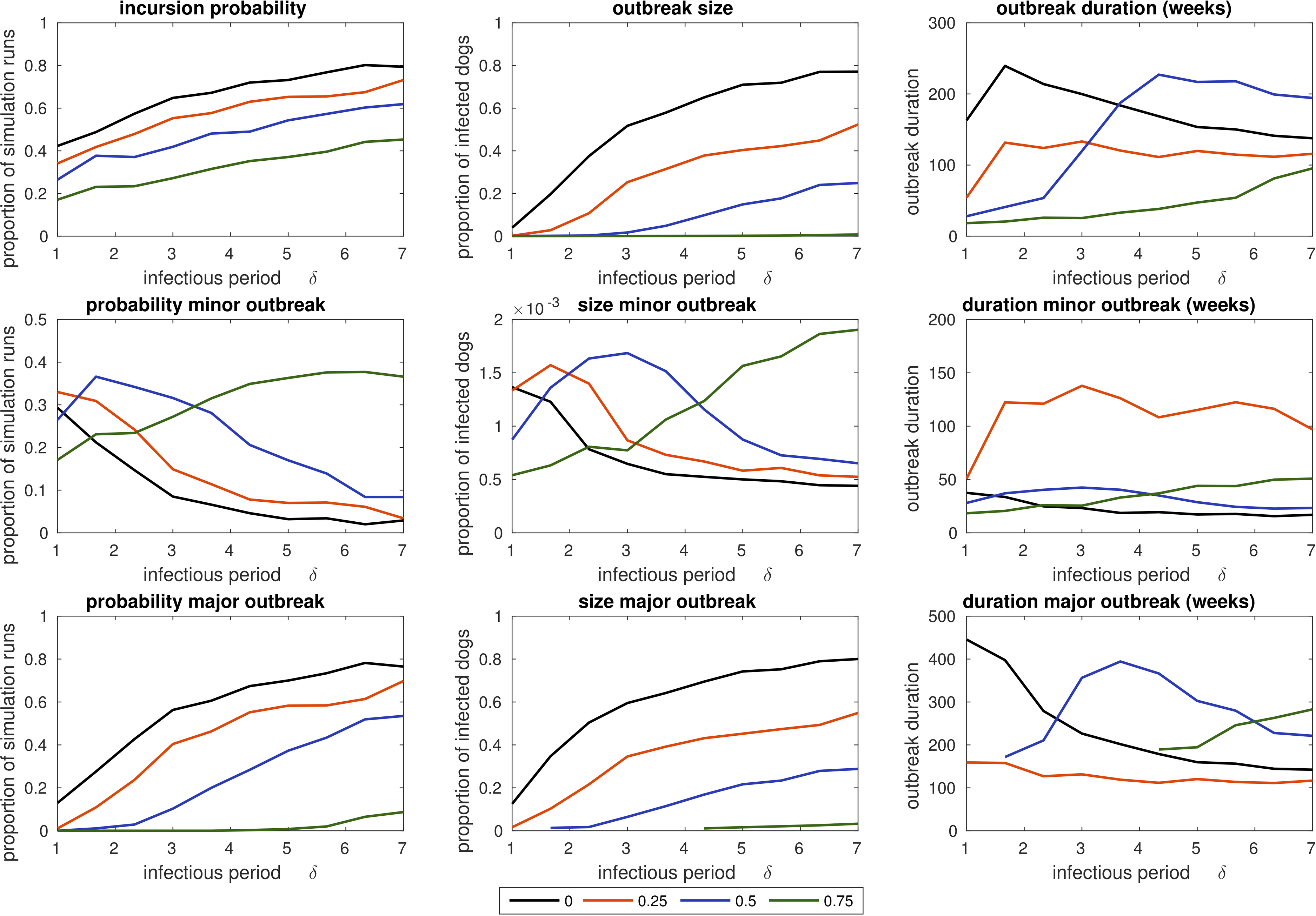

Supplement: S7 Fig — The colors correspond to different vaccination coverages. For each vaccination coverage and parameter value the mean of 1000 simulation runs is shown. Simulation runs where more than one dog gets infected are classified as incursion. Simulation runs where more than one dog and less than 1% of the population get infected are classified as minor outbreaks. Simulation runs where more than 1% of the population gets infected are classified as major outbreaks. Incursions include minor and major outbreaks. (TIF) [file pntd.0006680.s007.tif]
