# Supplementary material for: The importance of dog population contact network structures in rabies transmission
Source: PLoS Negl Trop Dis. 2018 Aug 1;12(8):e0006680. doi: 10.1371/journal.pntd.0006680 (PMC6089439; doi:10.1371/journal.pntd.0006680)
Supplement: S1 Table — (PDF) [file pntd.0006680.s008.pdf]

| Parameter | Usage | Description | Range | Type |
|---|---|---|---|---|
| $\kappa$ | network construction | scaling of spatial connection | [20,30] | discrete |
| $\tau$ | network construction | proportion of far roaming dogs | [0.5, 1] | continuous |
| $\lambda$ | network construction | mean number of peers of far roaming dogs | [20,30] | discrete |
| $\delta$ | transmission model | infectious period | [1,7] | discrete |
| $\sigma$ | transmission model | incubation period | [7, 730] | discrete |
| $\beta$ | transmission model | transmission rate | [0.015, 0.02] | continuous |
